# Mast cells exert pro-inflammatory effects of relevance to the pathophyisology of tendinopathy

**DOI:** 10.1186/ar4374

**Published:** 2013-11-08

**Authors:** Hayedeh Behzad, Aishwariya Sharma, Rouhollah Mousavizadeh, Alex Lu, Alex Scott

**Affiliations:** 1 Department of Physical Therapy, UBC, 2635 Laurel Street, Vancouver, BC V5Z 1M9, Canada

## Abstract

**Introduction:**

We have previously found an increased mast cell density in tendon biopsies from patients with patellar tendinopathy compared to controls. This study examined the influence of mast cells on basic tenocyte functions, including production of the inflammatory mediator prostaglandin E2 (PGE_2_), extracellular matrix remodeling and matrix metalloproteinase (MMP) gene transcription, and collagen synthesis.

**Methods:**

Primary human tenocytes were stimulated with an established human mast cell line (HMC-1). Extracellular matrix remodeling was studied by culturing tenocytes in a three-dimensional collagen lattice. Survival/proliferation was assessed with the 3-(4,5-dimethylthiazol-2-yl)-5-(3-carboxymethoxyphenyl)-2-(4-sulfophenyl)-2H-tetrazolium salt (MTS) assay. Levels of mRNA for *COX-2, COL1A1, MMP1*, and *MMP7* were determined by quantitative real-time polymerase chain reaction (qPCR). Cox-2 protein level was assessed by Western blot analysis and type I procollagen was detected by immunofluorescent staining. PGE_2_ levels were determined using an enzyme-linked immunosorbent assay (ELISA).

**Results:**

Mast cells stimulated tenocytes to produce increased levels of COX-2 and the pro-inflammatory mediator PGE_2_, which in turn decreased *COL1A1* mRNA expression. Additionally, mast cells reduced the type I procollagen protein levels produced by tenocytes. Transforming growth factor beta 1 (TGF-β1) was responsible for the induction of Cox-2 and PGE_2_ by tenocytes. Mast cells increased *MMP1* and *MMP7* transcription and increased the contraction of a three-dimensional collagen lattice by tenocytes, a phenomenon which was blocked by a pan-MMP inhibitor (Batimastat).

**Conclusion:**

Our data demonstrate that mast cell-derived PGE_2_ reduces collagen synthesis and enhances expression and activities of MMPs in human tenocytes.

## Introduction

Tendons are dense connective tissues, responsible for transmitting load between muscle and bone. The primary cell type in tendons, tenocytes, may be influenced by the presence of mast cells in their microenvironment. Although associations between mast cells and a number of chronic conditions including pulmonary fibrosis [1], renal fibrosis [2], and scleroderma [3] have been established, very few data are available on the possible link between mast cells and the failed tissue healing or fibrosis that is evident in chronically injured tendon tissues. We have previously documented a greater number of mast cells in the tendons of patellar tendinopathy subjects compared with healthy controls [4], and increased mast cell numbers have also been detected in the injured rotator cuff [5]. We also found that tendon overuse (intensive uphill treadmill running) led to an increased density of mast cells in the Achilles paratendon [6]. Certainly, since mast cells are present in and around tendons, and since they possess an arsenal of inflammatory mediators and growth factors, they could exacerbate certain features of tendon injury or overuse pathology such as inflammation, excessive cell proliferation, and inappropriate matrix remodeling, contributing to the formation of poorly organized repair tissue, and ongoing injury and pain.

In this study, we utilized an *in vitro* culture system using human primary tendon fibroblasts (tenocytes) and an established human mast cell line (HMC-1) to investigate potential effects of mast cells on tenocyte gene expression and function. Our data provide evidence for mechanisms by which mast cells could contribute to the development of tendinopathy, including a transforming growth factor beta (TGFβ)-dependent upregulation of cyclooxygenase (COX)-2, a reduction of *COL1A1* mRNA and type I procollagen protein levels in tenocytes, and a matrix metalloproteinases (MMP)-dependent increase in collagen remodeling activity. The current studies support the hypothesis that inflammatory cells may be involved in the development of tendon injury or overuse pathology [7].

## Methods

### Cell culture

Tendon fibroblasts (tenocytes) were isolated from human hamstring tendon, after obtaining informed consent from each subject. Ethics approval was obtained from the review board at the University of British Columbia.

Briefly, samples of healthy hamstring tendons from patients undergoing anterior cruciate ligament reconstruction were trimmed to remove fat and muscle, washed with phosphate-buffered saline (PBS), and enzymatically digested with filtered 1.5 mg/ml collagenase (Clostridopeptidase A; Sigma, Oakville, Ontario, Canada) in serum-free Dubecco’s modified Eagle medium (DMEM; HyClone, South Logan, Utah, USA) for 30 minutes at 37°C with shaking. Following collagenase digestion, 1× trypsin (TrypLE™ Select; Gibco, Life Technologies, Burlington, ON, Canada) was added for an additional 5 minutes. The cell–collagenase–trypsin mixture was then centrifuged at 1,200 rpm for 5 minutes and the resulting cell pellet was resuspended and cultured in DMEM supplemented with 10% fetal bovine serum (FBS), and 1% penicillin–streptomycin at 37°C in 5.0% carbon dioxide. The HMC-1 cell line was originally established from a patient with mast cell leukemia [8]. HMC-1 mast cells were grown in RPMI 1640 media (HyClone) supplemented with 10% FBS and l-glutamine at 37°C in 5.0% carbon dioxide.

### HMC-1 conditioned media and sonicate

HMC-1 cells (1.5 × 10^6^ cells/ml media) were cultured for 24 hours in the presence or absence of serum. Cells were centrifuged at 1,200 rpm for 5 minutes, and supernatant was collected as HMC-1 conditioned media. For the generation of HMC-1 sonicate (mast cell sonicate (MCS)), HMC-1 cells were cultured in the presence or absence of indomethacin (2 μM) for 48 hours, then washed with PBS and resuspended in serum-free DME/F-12 media (HyClone) at a concentration of 5 × 10^6^ cells/ml, and then sonicated on ice for 1 minute (duty cycle 5 seconds, output power 50%; Heat Systems Ultrasonics). The sonicated mixture was centrifuged at 13,000 rpm for 10 minutes at 4°C. Supernatants were collected and stored at −80°C.

### Experimental conditions

Tenocytes were grown to confluence in six-well plates. For quantitative polymerase chain reaction (qPCR) studies, cells were treated with 2 ng/ml TGF-β1 (R&D Systems, Minneapolis, MN, USA), 2 μM A83-01 (Tocris Biosciences, Minneapolis, MN, USA), or the HMC-1 conditioned media (prepared with 10% FBS) for 2, 4, 6, 8, and 24 hours. For western blot analyses, confluent tenocytes were grown in the absence of FBS overnight. Cells were then treated with TGF-β1, A83-01, and/or serum-free mast cell conditioned media for 2, 4, 6, and 24 hours.

### Cell viability

All of the inhibitors were tested for their potential toxic effects on cells by performing the Trypan Blue exclusion assay. For A83-01 and indomethacin, the lowest inhibitory concentration that provided maximum inhibition and full viability was used. Batimastat provided full viability at the concentrations used in this study.

### RNA isolation, cDNA synthesis, and real-time qPCR

Total RNA was isolated from cells using the Fermentas GeneJET™ RNA purification kit (Thermo Scientific, Ottawa, Ontario, Canada) as specified by the manufacturer. For qPCR, 1 μg total RNA was converted to cDNA using a High Capacity cDNA Reverse Transcription kit (Invitrogen, Burlington, ON, Canada). qPCR was performed with SYBR Green PCR Mix from Roche (Roche Applied Science, Laval, Quebec, Canada). Samples were run in triplicate to ensure reproducibility of the results. For internal control, *GAPDH* was used as the housekeeping gene. The forward and reverse primers are presented in Table 1.

**Table 1:** Sequences of the quantitative polymerase chain reaction primer sets used in this study

**Target**	**Primer sequence**
*COX-2*	Forward: 5′-CAGGGTTGCTGGTGGTAGGA-3′
	Reverse: 5′-GCATAAAGCGTTTGCGGTAC-3′
*Col1a1*	Forward: 5′-TGTTCAGCTTTGTGGACCTCCG-3′
	Reverse: 5′-CGCAGGTGATTGGTGGGATGTCT-3′
*MMP1*	Forward: 5′-AGCTGGGATATTGGAGCAGC-3′
	Reverse: 5′-TCCGCTTTTCAACTTGCCTTTG-3′
*MMP7*	Forward: 5′-GTCTCTGGACGGCAGCTATG-3′
	Reverse: 5′-GATAGTCCTGAGCCTGTTCCC-3′
*GAPDH*	Forward: 5′-TCTTTTGCGTCGCCAGCCGAG-3′
	Reverse: 5′-TGACCAGGCGCCCAATACGAC-3′

### Western blotting

Tenocytes were lysed in solubilization buffer (10% Tris–HCl pH 7.7, 10% Triton X-100, 10% glycerol, 2% NaCl, 0.005% ethylenediamine tetraacetic acid, protease and phosphatase inhibitors), sonicated, and centrifuged at 13,000 rpm for 10 minutes. Protein concentration was measured using the BCA Bicinchoninic acid assay (Thermo Scientific, Rockford, IL, USA). After boiling with the gel loading dye (10% glycerol, 62.5 mM Tris–HCl pH 6.8, 2% SDS, Bromophenol Blue), equal amounts of protein (40 to 50 μg) were separated by SDS-PAGE electrophoresis. The resolved proteins were transferred electrophoretically onto nitrocellulose membrane (Bio-Rad, Mississauga, Ontario, Canada) by wet transfer. The membrane was incubated for 45 minutes at room temperature with blocking solution (5% BSA, Tris base pH 7.4, 0.05% Tween-20) and then incubated with a 1:200 dilution of a goat polyclonal antibody to Cox-2 (C-20, sc-1745; Santa Cruz, Dallas, Texas, USA) overnight at 4°C. The membranes were washed in Tris-buffered saline, 0.05% Tween-20 and incubated with a horseradish peroxidase goat secondary antibody (Dako, Burlington, Ontario, Canada) at a 1:2,500 dilution in Tris-buffered saline, 0.05% Tween-20 for 1 hour at room temperature. After washing, proteins were incubated with an electrochemiluminescent detection kit (Supersignal® West Femto Maximum Sensitivity Substrate; Thermo Scientific, Rockford, IL, USA), and detected using Perkin Elmer’s Geliance 600 Imaging System and GeneSnap from SynGene software.

### Immunohistochemistry

Tenocytes were grown to confluence in an eight-well glass slide (Lab-Tek®II Chamber Slide™ System; Nalge Nunc International Corp., Rochester, NY, USA). Cells were either untreated or treated with HMC-1 conditioned media for 24 hours. For type I procollagen immunostaining, cells were fixed with 5% buffered formalin for 20 minutes, washed with PBS, blocked in blocking buffer (1% FBS and 0.2% Tween-20 in PBS) for 30 minutes, permeabilized with 0.4% Triton X-100 for 10 minutes, and then washed and incubated with 1:500 dilution of a mouse monoclonal antibody to type I procollagen (M-38-c; Developmental Studies Hybridoma Bank, University of Iowa, Iowa City, IA, USA) in blocking buffer for 2 hours. Cells were then washed and incubated with 1:500 dilution of the Alexa Fluor® 488 goat anti-mouse secondary antibody (Invitrogen) in blocking buffer for 30 minutes at room temperature in the dark. Nuclei were visualized with Hoechst (Invitrogen) diluted to 1:10,000 in PBS for 2 minutes at room temperature. Stained cells were washed, mounted using aqueous mounting media (Immu-mount™; Thermo Scientific), and viewed using a Zeiss Axio Observer A1 fluorescent microscope equipped with a Zeiss AxioCam Lcm1 camera. The Zeiss AxioVision SE64 Rel. 4.8 software was used for taking pictures. The fluorescent signal was quantified using the open source software CellProfiler [9] as described previously [10]. Briefly, the mean arbitrary fluorescence intensity unit of each cell was first calculated based on the fluorescent intensity of the total pixels within the cell. A total of 600 cells were analyzed for each condition, and the mean of mean arbitrary fluorescence intensity units were then calculated based on the total number of cells analyzed per condition.

### Enzyme-linked immunosorbent assay

Tenocytes were grown to confluence in 24-well plates. Cells were pretreated with A83-01 (2 μM) or indomethacin (2 μM) for 1 hour. Following pretreatment, cells were either left untreated or were treated with mast cell conditioned media in the presence or absence of the above inhibitors for 6 hours. Plates were washed three times with PBS, and 400 μl FBS-free DMEM was added to each plate for an additional 24 hours, following which the tenocyte conditioned media was collected and stored at −80°C for prostaglandin E_2_ (PGE_2_) enzyme-linked immunosorbent assay (ELISA). The PGE_2_ ELISA was performed using the Prostaglandin E_2_ EIA kit – Monoclonal (Cayman Chemical, Ann Arbor, MI, USA), according to the manufacturer’s instructions.

### Three-dimensional tenocyte-populated collagen matrix

PureCol (Bovine type I collagen solution, 3 mg/ml) was obtained from Advanced Biomatrix (San Diego, CA, USA). The tenocyte-populated collagen gel solution was prepared by mixing PureCol, 5× DMEM (pH 7.4), and tenocytes. The final concentration of each component in the mixture was 1× DMEM, 2.1 mg/ml PureCol, and 5 × 10^5^ tenocytes/ml. Gels were cast in 24-well plates. In each well, 450 μl of the above gel preparation (containing 2.5 × 10^5^ tenocytes) was mixed with either 50 μl serum-free DME/F-12, or 50 μl of the serum free MSC (extracted from 2.5 × 10^5^ mast cells). TGF-β1 (1 to 2 ng/ml), A83-01 (2 μM), indomethacin (2 μM), and batimastat (1, 2, or 5 μM) were directly added to the gel mixture before polymerization. Gels were allowed to polymerize at 37°C for 30 minutes and, once polymerized 500 μl serum-free DME/F-12 was added to each well. For the collagen gel contraction assay, the gels were incubated at 37°C overnight and were released the following day using sterile 20 μl pipette tips to go around the edges of the wells in order to detach the gels from the sides. Gels were scanned daily using an image scanner (V500; Epson) and the area of each gel was measured with image processing software (Image J, NIH, Bethesda, MD, USA). The data for each time point were expressed as percent area relative to the initial area at day 0.

For the *MMP1* and *MMP7* expression studies, RNA was extracted from the gels as follows. Gels were cast as above and cultured for 24 and 48 hours without being released. At the end of each time point, gels were snap frozen in liquid nitrogen, and stored at −80°C. The frozen samples were pulverized with a mikro-Dismembrator S (Sartorius, Mississauga, ON, Canada) at 3,000 rpm for 30 seconds and the RNA was extracted using Trizol reagent (Life Technologies, Mississauga, ON, Canada), and purified using the Fermentas GeneJET™ RNA purification kit (Thermo Scientific) as specified by manufacturer.

### The MTS assay

Tenocytes were serum starved overnight. Cells were either left untreated or treated with serum-free HMC-1 conditioned media for 24 and 48 hours. MTS/PMS reagent (Promega, Madison, WI, USA) was prepared in PBS at 1/0.92 mg/ml and was added at a ratio of 20:1 to cells and plates were incubated for 3 to 4 hours. The absorbance at 490 nm was then recorded with a spectrophotometer. The cell viability of treated cells relative to untreated cells was expressed as a percentage for each time point.

### Statistical analysis

Data were expressed as the mean ± standard error of the mean (SEM). The Mann–Whitney *U* test and Student’s *t* test were used to determine the significance of differences between two experimental groups. The collagen gel contraction assay was analyzed with two-way analysis of variance followed by Bonferroni’s *post-hoc* test. The effect of Batimastat on collagen matrix contraction was analyzed with a linear mixed model fit by restricted maximum likelihood. *P* <0.05 was considered significant. Differences in PGE_2_ production by tenocytes exposed to mast cell conditioned media with/without inhibitors (indomethacin, A83-01) were examined using one-way analysis of variance followed by Bonferroni’s *post-hoc* test with an overall alpha level set at *P* = 0.01.

## Results

**Figure 1:**
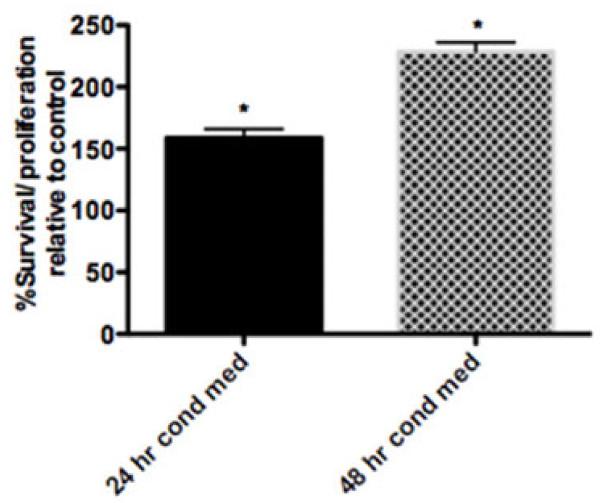
**Mast cells promote the survival/proliferation of human tenocytes.** Cells were cultured in the presence or absence of HMC-1 conditioned media (cond med) for 24 and 48 hours. The MTS assay revealed a time-dependent effect of mast cells on the survival/proliferation of tenocytes. Bars show the mean ± standard error of the mean of three experiments. Mann–Whitney *U* test, *P <0.001 relative to control.

Tendinopathy is associated with increased fibroblast hypercellularity [11]. Initial experiments therefore examined the influence of mast cells on tenocyte survival/proliferation. The MTS assay revealed that mast cells significantly enhanced the survival/proliferation of human tenocytes. Tenocytes treated with HMC-1 conditioned media for 24 or 48 hours showed a significant increase in cell survival/proliferation over untreated control samples (Figure 1). The mean ± SEM percent survival/proliferation in HMC-1 conditioned media treated relative to control samples at 24 hours was 158.8 ± 2.4% and at 48 hours was 226.6 ± 2.49%. HMC-1 sonicate also enhanced the survival/proliferation of tenocytes, albeit to a lesser degree (data not shown).

**Figure 2:**
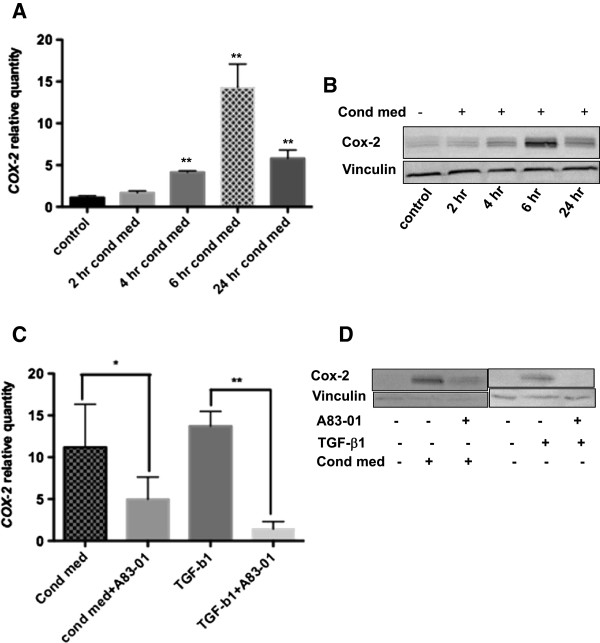
**Transforming growth factor beta-1 contributes to a mast cell-induced increase in COX-2 mRNA and protein expression by human tenocytes.** Tenocytes were cultured in the presence or absence of HMC-1 conditioned media (cond med), recombinant active transforming growth factor beta-1 (TGF-β1), and A83-01 for the indicated times. Quantitative polymerase chain reaction and western blots show increased cyclooxygenase *COX-2* mRNA **(A)** and protein **(B)** in response to HMC-1 conditioned media. A83-01 reduced *COX-2* mRNA **(C)** and Cox-2 protein **(D)** by tenocytes in response to treatment with mast cell conditioned media or TGF-β1 for 6 hours. Data represent mean ± standard error of the mean of at least three independent experiments, each run in triplicate. Mann–Whitney *U* test. *P = 0.022, **P = 0.0037.

Patellar tendinopathy tissue expresses increased levels of *COX-2* compared with controls [12]. We have found by qPCR analysis and western blotting that HMC-1 conditioned media significantly enhanced the mRNA and protein expression of Cox-2 in monolayer cultures of human tenocytes in a time-dependent manner (Figure 2A and 2B, respectively). The Cox-2 levels peaked at 6 hours relative to control, and declined by 24 hours. At 6 hours, the *COX-2* mRNA mean ± SEM quantity relative to control (RQ) was 14.29 ± 1.146 and the Cox-2 protein mean ± SEM fold-change relative to control was 5.119 ± 0.302. The TGF-β1 receptor ALK5 kinase inhibitor, A83-01 (2 μM), significantly blocked *COX-2* mRNA (mean ± SEM RQ 4.982 ± 0.8868) and Cox-2 protein expression (mean ± SEM fold-change 2.300 ± 0.5147, P = 0.037) by tenocytes in response to HMC-1 conditioned media as shown in Figure 2C and 2D, respectively. As expected, exogenous active human TGF-β1 (2 ng/ml) enhanced *COX-2* expression by tenocytes, which also peaked at 6 hours (data not shown). Additionally, the *COX-2* mRNA and Cox-2 protein expression in response to exogenous TGF-β1 were almost entirely inhibited by A83-01 (Figure 2C,D).

**Figure 3:**
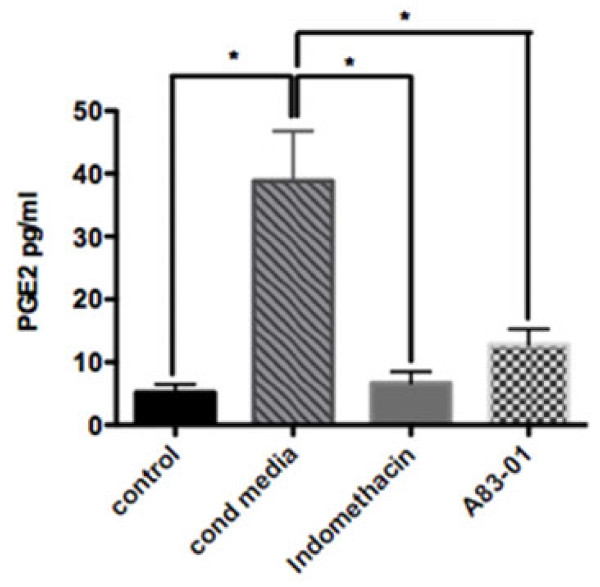
**Mast cell-induced prostaglandin E**_**2 **_**production by human tenocytes is mediated through transforming growth factor beta-1.** Tenocytes were stimulated with HMC-1 conditioned media (cond media) with or without indomethacin (2 μM) or A83-01 (2 μM) for 6 hours. Media were removed, cells were washed three times with phosphate-buffered saline, and incubated with a fresh serum-free media for 24 hours. Enzyme-linked immunosorbent assay measured the prostaglandin E_2_ (PGE_2_) concentration in tenocyte-conditioned media. Data represents mean ± standard error of the mean of at least three separate experiments. One-way analysis of variance, *P* <0.001. *Statistically significant Bonferroni *post-hoc* tests of *P* <0.01.

Since Cox-2 is one of the major enzymes contributing to the production of PGE_2_ and since PGE_2_ is a major inflammatory mediator in tendon [13], we used ELISA to measured PGE_2_ levels in the conditioned media of tenocytes stimulated with mast cells. As shown in Figure 3, treatment of tenocytes with the HMC-1 conditioned media significantly enhanced the production of PGE_2_ by human tenocytes compared with control untreated tenocytes (mean ± SEM 38.85 ± 3.951 pg/ml vs. 5.200 ± 0.6364 pg/ml). This increase in PGE_2_ production was shown to be dependent on both TGF-β1 and cyclooxygenase, since both A83-01 and indomethacin significantly reduced PGE_2_ production by tenocytes in response to treatment with mast cell conditioned media (mean ± SEM 12.83 ± 1.226 pg/ml and 6.650 ± 0.9078 pg/ml, respectively), as shown in Figure 3.

**Figure 4:**
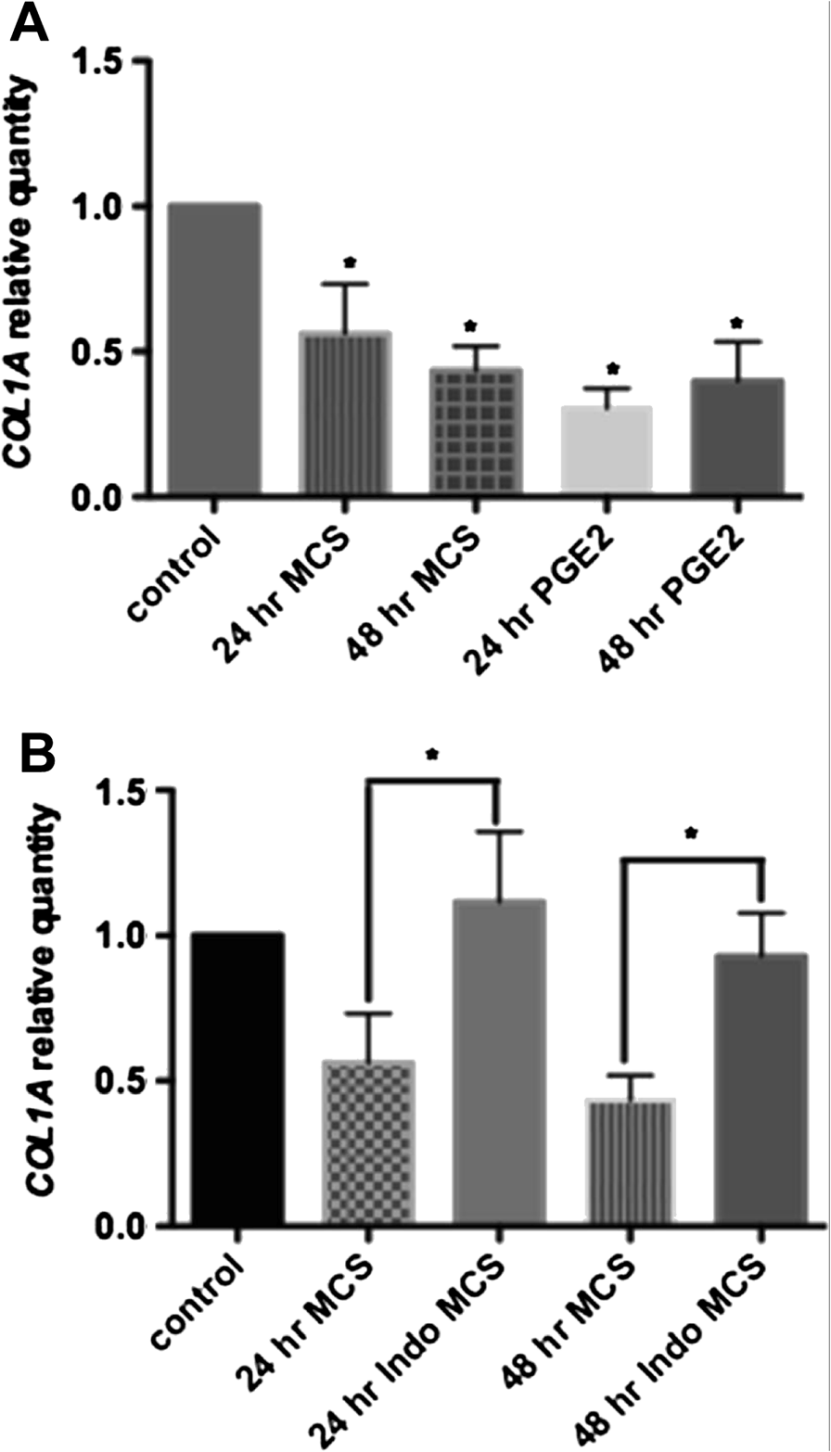
**Mast cell-induced modulation of collagen expression by human tenocytes is mediated through prostaglandin E**_**2**_**.** Tenocytes cultured in a three-dimensional collagen matrix were stimulated with mast cell sonicate (MCS), prostaglandin E_2_ (PGE_2_, 5 μM), or sonicate generated from indomethacin-treated mast cells (Indo MCS). Quantitative polymerase chain reaction analysis shows *COL1A1*transcription levels by tenocytes in response to MCS and PGE_2_**(A)**, and MCS versus Indo MCS **(B)**. *P <0.001.

**Figure 5:**
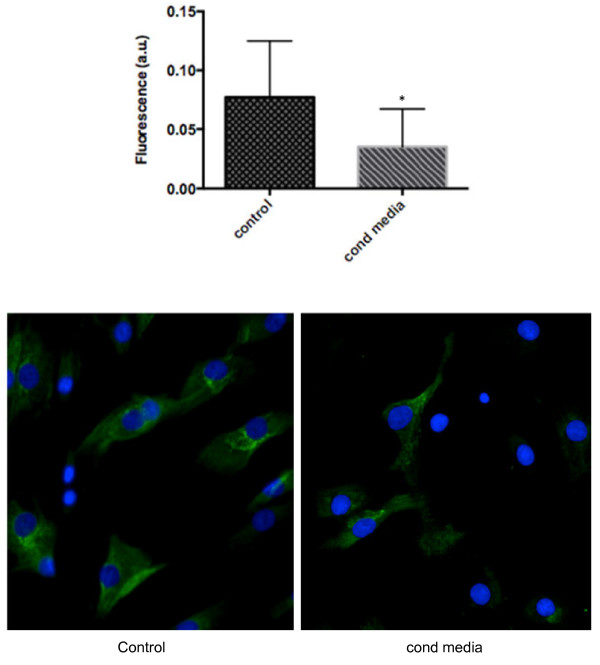
**Mast cells reduce type I procollagen protein expression by tenocytes.** Immunofluorescent staining for type I procollagen protein in a monolayer of tenocytes stimulated with HMC-1 conditioned media (cond media) for 24 hours. The immunofluorescent images were analyzed for intensity of procollagen immunoreaction using the open source software CellProfiler and data are presented as mean arbitrary fluorescence intensity units (a.u.). Representative immunofluorescent images of control versus conditioned media-treated tenocytes are shown. Data represent mean ± standard error of the mean of at least three separate experiments, each run in triplicate. Mann–Whitney *U* test, *P <0.001.

PGE_2_ has been shown to downregulate type I collagen expression [14] and tendinopathic or injured tendons demonstrate a lower concentration of type I collagen than healthy tendon, and a reduced load-bearing capacity [15]. Our qPCR analysis revealed that addition of HMC-1 MCS to tenocytes embedded in a collagen matrix significantly reduced *COL1A1* mRNA by tenocytes relative to untreated cells, as shown in Figure 4A (mean ± SEM RQ 0.5624 ± 0.04386 at 24 hours and 0.4356 ± 0.02135 at 48 hours). Exogenous addition of PGE_2_ (5 μM) to tenocytes in the three-dimensional collagen matrix independently reduced *COL1A1* expression by tenocytes relative to control, both at 24 hours (mean ± SEM RQ 0.3065 ± 0.02276) and at 48 hours (mean ± SEM RQ 0.3999 ± 0.04437) as shown in Figure 4A. Next, we examined the role of PGE_2_ in mast cell-mediated downregulation of *COL1A* mRNA. We show that under conditions where production of PGE_2_ both in mast cells and tencytes were substantially blocked with indomethacin, Indo MCS (the sonicate generated from indomethacin-treated mast cells) did not have any effect on *COL1A1* expression by tenocytes relative to control untreated cells (refer to Methods for how Indo MCS was generated). The mean ± SEM *COL1A1* RQ was 1.116 ± 0.0802 at 24 hours and 0.9298 ± 0.04274 at 48 hours, as shown in Figure 4B. Immunofluorescent staining for type I procollagen (Figure 5) revealed that tenocytes treated with mast cell conditioned media for 24 hours produced significantly less type I procollagen compared with control tenocytes. Mean arbitrary fluorescence intensity units were 0.03541 ± 0.0011 for cells treated with HMC-1 conditioned media versus 0.07719 ± 0.0019 for control samples. A representative immunofluorescence image is shown in Figure 5. These data demonstrate that HMC-1 mast cells modulate collagen expression by tenocytes through PGE_2_-mediated events.

**Figure 6:**
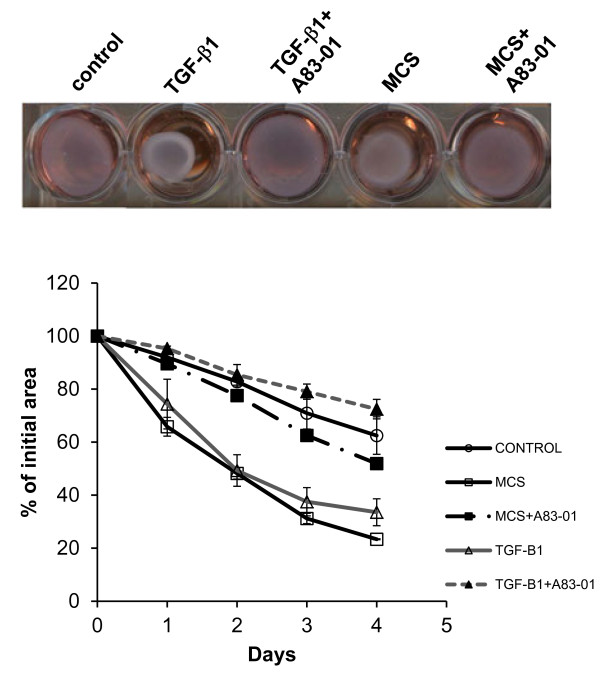
**Mast cell-induced contraction of a three-dimensional collagen matrix is mediated through transforming growth factor beta-1.** Tenocytes embedded in a three-dimensional collagen gel were stimulated with mast cell sonicate (MCS) or active transforming growth factor beta-1 (TGF-β1), in the presence or absence of A83-01. Data represent mean ± standard error of the mean of at least three separate experiments, each run in triplicate. Two-way analysis of variance followed by Bonferroni’s *post-hoc* test was used for comparisons. *P* <0.001 for days 2 to 4 for the following group comparisons: control versus MCS, MCS versus MCS + A83-01, control versus TGF-β1, and TGF-β1 versus TGF-β1 + A83-01.

*In vitro*, contraction of the three-dimensional collagen matrix by fibroblasts has been widely used as a model system to measure contractile activity of fibroblasts [16]. Using this model, we found that MCS significantly enhanced the contraction of a three-dimensional collagen lattice in a time-dependent manner (Figure 6). HMC-1 mast cells and the HMC-1 conditioned media also enhanced contraction in the same system (data not shown). Exogenous recombinant active human TGF-β1 independently enhanced tenocyte-mediated collagen matrix contraction in a time-dependent manner (Figure 6). Inhibition of the TGF-β1 activity with A83-01 (2 μM) almost completely abrogated the increase in matrix contraction in response to MCS and TGF-β1. These findings indicate that mast cell-derived TGF-β1 mediates collagen remodeling by tendon fibroblasts.

**Figure 7:**
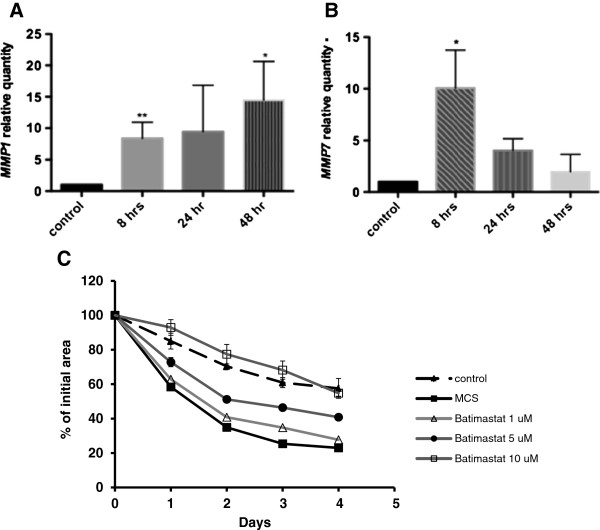
**Matrix metalloproteinases contribute to the mast cell-induced contraction of tenocyte populated collagen matrices.** Tenocytes embedded in collagen matrices were stimulated with mast cell sonicate (MCS) for the indicated times and RNA was extracted. The quantitative polymerase chain reaction analysis shows the mRNA expression of matrix metalloproteinases *MMP1***(A)** and *MMP7***(B)** by tenocytes in response to MCS. Data represent mean ± standard error of the mean of at least three separate experiments, each run in triplicate (Student’s *t* test, *P <0.025 relative to control). **(C)** A pan-MMP inhibitor (batimastat) added to the collagen gel inhibited contraction of the collagen matrix in response to MCS in a concentration-dependent and time-dependent manner (*P* <0.001 for effects of both time and treatment).

MMPs play significant roles in tissue remodeling and wound healing [17,18]. We found that MCS significantly enhanced the expression of *MMP1* and *MMP7* in human tenocytes (Figure 7A,B). Although exogenous addition of active human TGF-β1 to tenocyte culture independently enhanced *MMP7* mRNA expression by tenocytes, A83-01 did not reduce the observed increase in *MMP1* or *MMP7* expression in response to HMC-1 sonicate (data not shown). However, blocking MMP activities with a pan-MMP inhibitor, batimastat, significantly inhibited mast cell-mediated contraction of the three-dimensional collagen lattice in a concentration-dependent and time-dependent manner as shown in Figure 7C. These results suggest that the observed increase in matrix remodeling in response to HMC-1 sonicate is due in part to induction of MMP activities.

## Discussion

Mast cells are significant players in many degenerative and fibrotic disorders, with the capacity to directly influence fibroblasts, which coordinate the production and remodeling of the extracellular matrix. In the current study, we explored whether mast cells could contribute to pathophysiological processes known to occur in tendinopathy, including expression of proinflammatory or fibrotic substances [19], tendon hypercellularity [20], and remodeling or degradation of the extracellular matrix [21]. We found that human mast cells enhanced tenocyte survival/proliferation and increased their production of the inflammatory mediator PGE_2_, which decreased the production of type I collagen by tendon fibroblasts. In addition, we found that mast cells enhanced the contraction and remodeling of a three-dimensional collagen matrix by tenocytes, processes that were mediated by TGF-β1 and MMPs. Since these mechanisms have been implicated in the pathophysiology of tendinopathy, our results may be of relevance to understanding the development of this condition.

Increased fibroblast proliferation has been reported in a number of different fibrotic disorders, and mast cells have been shown to contribute to this phenomenon in the skin, lung and other organs [22]. We have shown in this study that HMC-1 conditioned medium enhanced the survival/proliferation of human tenocytes, a cell type whose density is substantially increased in chronically injured tendon [23]. This effect of mast cells on tenocytes was not unexpected, since mast cells carry a number of different mediators and growth factors that could potentially enhance cell proliferation through several mechanisms, including mast cell tryptase [24], heterotypic cell–cell adhesion and interleukin-4 [25], and histamine [26].

Enhanced expression of *COX-2* and increased PGE_2_ have been detected in tendinopathy [27]. Although mechanical loading of tendon is believed to enhance the expression of *COX-2* and the subsequent production of PGE_2_ by tendon cells [28], the role of inflammation *per se* as a contributing factor to these events is controversial [29]. Our finding that human mast cells enhanced the expression of Cox-2 and increased the production of PGE_2_ in tendon fibroblasts is in line with previous results using a variety of human fibroblast cell types [30]. Mast cells contain various growth factors, cytokines, and proteases that could potentially mediate Cox-2 production by fibroblasts, and TGF-β1 is one such cytokine. Here we have shown that mast cell-derived TGF-β1 plays a significant role in enhancing the production of the inflammatory mediator PGE_2_ by human tenocytes. Tenocytes treated with an inhibitor (A83-01) of the TGF-β1 receptor (ALK5) showed a significant reduction in *COX-2* expression and PGE_2_ production in response to mast cells. We know from our own unpublished observations and those of others that HMC-1 mast cells constitutively express TGF-β1mRNA [31] and that the TGF-β1protein is constitutively present in the conditioned media of HMC-1 cells. Although mast cells contain other inflammatory mediators that could enhance Cox-2 production by fibroblasts [30], our findings implicate TGF-β1 as a mast cell-derived cytokine, which significantly enhanced the production of Cox-2 and PGE_2_ by tendon fibroblasts. This increase in PGE_2_ production in response to mast cells could adversely affect collagen synthesis and the subsequent healing response in tendons.

Understanding the production and remodeling of the extracellular matrix, in particular collagen, is of relevance to the etiology of degenerative or fibrotic disorders such as tendinopathy. We have shown here that HMC-1 sonicate significantly reduced *COL1A1* mRNA transcription and type I procollagen protein in tenocytes, both in a three-dimensional collagen matrix and in a monolayer cell culture. This result is in line with the findings that MSC reduced collagen synthesis by peritoneal and lung fibroblasts [32]. In contrast to our findings, Gruber and colleagues showed that MSC enhanced *COL1A1* expression by skin fibroblasts [33]. Differences in the experimental conditions and heterogeneity in fibroblast responses could perhaps explain the different findings in the two studies.

Mast cells are known to play a significant role in tissue remodeling, healing, and fibrosis through their effect on fibroblasts. The three-dimensional fibroblast-populated collagen matrix has been widely used as a model system to study the contractile remodeling of extracellular matrix by fibroblasts during tissue healing. In this study, we show that the addition of MSC to a tenocyte populated collagen lattice significantly enhanced collagen matrix contraction. Our results are consistent with those of others who also found that mast cells or MSC significantly enhanced fibroblast-mediated contraction of a three-dimensional collagen matrix [22]. Mast cell tryptase [34], MMPs [35], intercellular gap junctions [36], and SCF/c-Kit heterotypic cell–cell interactions [37] are amongst the various mechanisms suggested to be involved in this process. However, we have found that TGF-β1 is a major player in mediating contraction of the tenocyte populated collagen matrix by HMC-1 cells, since blocking of the TGF-β1 receptor with A83-01 almost entirely inhibited matrix contraction.

The contraction of collagen matrix by fibroblasts is often assumed to result from the differentiation of cells into myofibroblasts, as gauged by their increased expression of contractile smooth muscle actin [38]. Surprisingly, in our system there was no increase in alpha smooth muscle actin in response to MSC/conditioned media or TGF-β1 (data not shown), despite the fact that these substances increased the rate of collagen matrix contraction. This prompted us to examine whether the increased collagen matrix contraction in response to HMC-1 could be due to increased expression of MMPs that are known to play a role in tendinopathies, including MMP1 [39] and MMP7 [40]. We found that both *MMP1* and *MMP7* mRNA in tenocytes were increased in response to MSC. MMP1 plays an important role in breaking down the extracellular matrix, and it specifically breaks down the interstitial type I, type II, and type III collagens. Enhanced *MMP1* expression could thus lead to matrix remodeling and collagen matrix contraction. The collagen receptor α_2_β_1_ has been linked to the increase in the induction of MMP1 and collagen matrix contraction in cultures of primary human fibroblasts [41]. Although exogenous active TGF-β1 in our hands enhanced *MMP7* transcription by tenocytes, the increase in *MMP1* and *MMP7* mRNA in response to mast cells was not mediated through TGF-β1. It has been shown previously that MMP7 can enhance the bioavailability of TGF-β1 in the extracellular matrix by releasing TGF-β1 from its bound complex with decorin [42]. A mast cell-mediated increase in *MMP7* expression and activity may thus have led to the increased TGF-β1-induced collagen matrix contraction. Other MMPs including MMP2 and MMP3 have also been reported to enhance the activity of mast cell-derived TGF-β1, leading to contraction of collagen matrix [35]. Similar to the findings of Margulis and colleagues [35], we have found that batimastat inhibited mast cell-mediated contraction of collagen gel, prompting us to conclude that MMP activity played a role in this process.

Mast cells have been linked to many degenerative conditions but currently no data are available on a possible role that mast cells might play in tendinopathy, although increased mast cell numbers have been detected in tendon biopsies from patients with tendinopathy. Although mast cells have been reported to affect the function of different fibroblast cells throughout the body, we are the first to report effects of mast cells on tendon fibroblast gene expression and functions. It is important to acknowledge, however, similar to any *in vitro* studies, that our findings need to be confirmed with further studies on clinical tendon specimens; co-localizing mast cells with tenocytes, for example, in the tendon microenvironment and analyzing the gene expression by tenocytes in these intact tendon tissues could further strengthen our findings. In addition, a mouse model that lacks mast cells would provide the ability to examine a possible influence of mast cells on tendon structural integrity and function.

In short, the present studies outline potential mechanisms by which mast cells could contribute to the pathogenesis of tendinopathy, and implicate the involvement of TGF-β1, PGE_2_, and MMPs (notably MMP1 and MMP7) in the response of tenocytes to mast cells. Enhanced tenocyte survival/proliferation, increased PGE_2_ production leading to downregulation of type I collagen, and increased MMP production and activity are amongst the potential mechanisms by which mast cells could modulate tendon degeneration and repair mechanisms. These findings suggest that therapies aimed at inhibiting mast cells in tendon could be beneficial in promoting recovery from tendon injury or tendinopathy.

## Conclusion

These results demonstrate that inflammatory processes mediated by mast cells regulate the function of human tenocytes leading to reduction in collagen production and increase in MMP expression by these cells. Overall, these data, combined with our previous finding of increased mast cell number in tendon biopsies from patients with patellar tendinopathy, suggest that mast cells have a potential to mediate matrix remodeling of tendon and contribute to tendon degradation. Further studies are required to demonstrate a direct role of mast cells in this process.

## Abbreviations

BSA: Bovine serum albumin; COX: Cyclooxygenase; DMEM: Dubecco’s modified Eagle medium; ELISA: Enzyme-linked immunosorbent assay; FBS: Fetal bovine serum; HMC-1: Human mast cell line 1; MCS: Mast cell sonicate; MMP: Matrix metalloproteinase; MTS: 3-(4,5-dimethylthiazol-2-yl)-5-(3-carboxymethoxyphenyl)-2-(4-sulfophenyl)-2H-tetrazolium salt; PBS: Phosphate-buffered saline; PGE2: Prostaglandin E2; qPCR: Quantitative polymerase chain reaction; RQ: Quantity relative to control; SEM: Standard error of the mean; TGFβ: Transforming growth factor beta.

## Competing interests

The authors declare that they have no competing interests.

## Authors’ contributions

HB designed and carried out experiments and data analysis. ASh carried out and analyzed qPCR experiments. RM designed and carried out human tenocyte culture experiments and protocols. AL conducted image analysis. ASc contributed to experimental design and obtained research funding. ASc is the guarantor. HB and ASc drafted the manuscript. All authors read and approved the final manuscript.

## Authors’ information

HB is a Research Associate at the University of British Columbia. ASh was a Research Assistant working in ASc’s laboratory. RM is a PhD student in Experimental Medicine. AL is the recipient of an Undergraduate Student Research Assistant award from the National Sciences and Engineering Research Council. ASc is an Assistant Professor at the University of British Columbia.
